# Alterations of Gut Bacteria in Hirschsprung Disease and Hirschsprung-Associated Enterocolitis

**DOI:** 10.3390/microorganisms9112241

**Published:** 2021-10-28

**Authors:** Sireekarn Chantakhow, Jiraporn Khorana, Kanokkan Tepmalai, Nonglak Boonchooduang, Nipon Chattipakorn, Siriporn C. Chattipakorn

**Affiliations:** 1 Department of Surgery, Division of Pediatric Surgery, Faculty of Medicine, Chiang Mai University, Chiang Mai 50200, Thailand; karnsire196@gmail.com (S.C.); nanji22@gmail.com (J.K.); kan_whan@yahoo.com (K.T.); 2 Clinical Epidemiology and Statistical Statistic Center, Faculty of Medicine, Chiang Mai University, Chiang Mai 50200, Thailand; 3 Department of Pediatrics, Division of Developmental and Behavioral Pediatrics, Faculty of Medicine, Chiang Mai University, Chiang Mai 50200, Thailand; nonglak.b@cmu.ac.th; 4 Cardiac Electrophysiology Unit, Cardiac Electrophysiology Research and Training Center, Faculty of Medicine, Chiang Mai University, Chiang Mai 50200, Thailand; 5 Neurophysiology Unit, Cardiac Electrophysiology Research and Training Center, Faculty of Medicine, Chiang Mai University, Chiang Mai 50200, Thailand; 6 Department of Oral Biology and Diagnostic Sciences, Faculty of Dentistry, Chiang Mai University, Chiang Mai 50200, Thailand

**Keywords:** Hirschsprung, aganglionosis, enterocolitis, gut microbiota, microbiome

## Abstract

Hirschsprung-associated enterocolitis (HAEC) is a common life-threatening complication of Hirschsprung disease (HSCR). It has been proposed that gut microbiota, which have an essential role in gut-homeostasis, are associated with HAEC. Recent studies demonstrated an increase in alpha diversity of fecal microbiota over time in HSCR mice and a decrease in diversity after surgery. In addition, clinical studies have reported a reduction in bacterial richness in HSCR children after surgery. Some studies revealed a difference in microbiota between the proximal ganglionic and distal aganglionic intestine and found a difference in bacterial character between fecal and colonic specimens. HAEC studies found an increase in *Proteobacteria*, especially *Escherichia* and *Enterobacteriaceae*, with a decrease in *Firmicutes* and *Bifidobacterium* in HAEC patients. However, the direction of alpha diversity in HAEC patients is still controversial. The self-comparison of microbiota in treatment periods suggested that probiotics might improve gut dysbiosis and decrease the frequency of enterocolitis, but some reported contradictory findings. This review comprehensively summarizes and discusses key findings from animal and clinical data of the distinct microbiome associated with HCSR and the association of gut dysbiosis with the development of HAEC. This information should be useful in the establishment of novel interventions to improve gut dysbiosis and prevent enterocolitis in HSCR patients.

## 1. Introduction

Hirschsprung disease (HSCR) is a congenital malformation of the enteric nervous system, characterized by the absence of ganglion cells in the distal intestine, resulting in spastic contractions at the affected bowel, and functional obstruction of the intestine above the aganglionic levels [1]. The worldwide incidence of HSCR is approximately 1 in 5000 live births with various spectrums of defective involvements, clinical manifestations, and outcomes of treatment [1,2]. Up to now, the only treatment of choice for HSCR is surgery by resection of an aganglionic bowel segment and reconstruction of the normally innervated intestine to the anus [1,3]. Currently, the surgical management for HSCR significantly improves symptoms and prolongs patient life; however, the surgery does not always equate to curative therapy. About one-third of patients with HSCR still develop postoperative complications [4], including obstructive symptoms, fecal soiling, and Hirschsprung-associated enterocolitis (HAEC) [1]. HAEC is a common life-threatening complication with about 5% mortality in HSCR patients [3]. HAEC is a severe inflammation of the intestine, and patients with HAEC will present with abdominal distension, vomiting, fever, and the passage of foul-smelling or bloody stools. The overall incidence of HAEC is 25–35% [4], and 25–37% of HSCR patients can develop HAEC after definitive treatment [2]. The etiology of HAEC is unclear. Some studies proposed underlying mechanisms behind HAEC, including decreased intestinal blood flow from marked and prolonged bowel dilation, impaired epithelial barrier function, bacterial translocation, gut immune dysfunction, and dysbiosis of gut microbiota [2,3,4].

Recent studies in both animal and clinical studies proposed that the alterations in gut microbiota or gut dysbiosis contribute to several diseases, including HSCR. Previous studies reported the various alterations of gut microbiota in both animal and clinical studies of HSCR and several studies indicated that gut dysbiosis might be associated with the development of enterocolitis [5,6,7,8,9]. However, the knowledge concerning gut microbiota in HSCR and HAEC is still limited. Considering the key role of gut microbiota in HSCR and HAEC, this review aims to summarize and discuss all evidence concerning the association between the alterations of gut microbiota and HSCR, as well as HAEC from animal studies and clinical studies. Any contradictory findings are also included and debated.

## 2. The Association of Gut Microbiota with Human Gut Maturation, Immunity, and Mucosal Defense Mechanism

The gut microbiome is a dynamic and distinct microbial ecosystem within the alimentary system of humans. The development of the gut microbiota in humans occurs after birth, and these gut microbiomes have been associated with many factors such as a mode of delivery, gestational age, perinatal antibiotic exposure, and have also been shown to change over time. At three years old, the microbiota changes to become similar to adult microbiota [10]. The other major factors which affect gut microbiota include race, individual diets, antibiotic exposure, and environment [11]. Under normal physiological conditions, the human gut provides the habitat and nutrients for the microbiota, while the microbiota promotes gut maturation, the immune system, and defense mechanisms of their host, via their metabolites such as vitamins, tryptophan, and short-chain fatty acids (SCFAs). In the host gut immune system, the microbiota metabolites such as SCFAs (butyrate) and tryptophan can enhance gut integrity, stimulate innate lymphoid cells group 3 (ILC3) to produce Interleukin-22 (IL-22), promote differentiation of naïve CD8 to CD4 T cells, regulate the expression of pro-inflammatory cytokines, including Interleukin-6 (IL-6), Interleukin-12 (IL-12), and tumor necrosis factor-α (TNF-α), and induce IgA secretion to handle inflammation [12,13]. The gut microbiome also plays a role in the reduction in the intestinal permeability, and an increase in the epithelial defense mechanism by creating a mucosal barrier and epithelial proliferation [11,12,13].

## 3. The Comparison of the Diversity of Gut Microbiota between Subjects with Hirschsprung Disease and Healthy Subjects: Evidence from Animal to Clinical Studies

Two animal studies [9,14] investigated the changes in gut microbiota in genetically modified HSCR mice. In 2014, Pierre et al. found a continuous increase in the alpha diversity of fecal microbiota over time in Ednrb knock-out mice, while the beta diversity in late-aged HSCR mice was significantly different when compared with the control wild-type mice [9]. Ward et al. in 2012 also demonstrated that the alpha diversity of the gut microbiota was higher in the mutant mice than that of wild-type mice, and the differences in alpha diversity between groups increased with age [14]. The authors also found a significant difference in beta diversity between colonic and fecal microbiota. These findings indicated the differences in microbiota between different specimens [14]. Another study in HSCR piglets found a significant difference in the beta diversity between HSCR and control groups [15]. The partial intestinal obstruction primarily presents in untreated Hirschsprung animals and patients. In 2018 Hegde et al. detected an increased alpha diversity of gut microbiome in rats with partial colonic obstruction [16]. This finding was consistent with the results in a model of functional GI disorder such as constipation [17]. This evidence suggests that increased diversity in bowel obstruction might result from the slower transit rate in the obstructed intestine [16].

In the periods of postoperative surgery, Cheng Z et al. performed microsurgical pull-through surgery in Ednrb knock-out mice [6]. They reported a decrease in alpha diversity of fecal microbiota in HSCR mice, when compared with wild-type mice after surgery. In wild-type mice, they found a difference in the beta diversity of the microbiota between each postoperative and preoperative period. The authors concluded that the diversity of gut microbiota decreased in HSCR after surgery, while the surgery itself might alter bacterial communities [6]. All these findings from animal studies indicated that the diversity in gut microbiota did not only differ between HSCR animals and wild-type animals, but also that the differences in the diversity could be observed between fecal and colonic microbiota. In addition, the alpha diversity of microbiota in the HSCR mice was higher than the wild-type mice, and it increased over time in line with age. That diversity was decreased post-surgical therapy.

Only one cross-sectional study of HSCR patients showed a decrease in the richness of gut microbiota in children with HSCR after definitive surgery, when compared with a control group of children [18]. The results from that study were similar to the post-surgical mice [6]. When considering previous postoperative studies of gut microbiota in patients, several authors proposed that there were alterations in gut microbiota after surgery, when compared with the preoperative period and in healthy adults [19,20,21,22]. Several studies detected changes of gut microbiota diversity after bariatric surgery in obese adults [19,20,21]. Following colorectal resection of patients with colorectal cancer, the study of Cong J et al. reported a significant decrease in the alpha diversity of gut microbiota after rectal resection when compared with the preoperative periods and in healthy adult controls [22]. This operation for rectal cancer most resembled the pull-through operation in HSCR patients, and the diversity of microbiota also decreased in a similar fashion after both operations [6,18]. These findings suggested that surgery, especially the rectal resection, itself induced the changes in bacterial communities.

Hirschsprung disease presents a varied spectrum of aganglionic involvement necessitating various resection lengths in surgical therapy. Pini Prato and colleagues reported gut microbiota of HSCR patients with total colonic aganglionosis (TCSA) comparing with rectosigmoid aganglionosis (RSA) [23]. The authors found a decrease in alpha diversity, and a significant difference in beta diversity in the fecal microbiota of TCSA patients, when compared with RSA after colonic resection. These findings suggested that entire-colon resection in the treatment of TCSA changed the intestinal microbiota [23]; therefore, the length of colonic resection might be one of the factors which affect the homeostasis of the gut microbiota. All of these findings are summarized in Table 1.

## 4. The Alterations of Taxa in Gut Microbiota between Subjects with Hirschsprung Disease and Healthy Controls: Evidence from Animal to Clinical Studies

The majority of HSCR studies in animals found a significant increase in *Proteobacteria* and Bacteroidetes with a decrease in *Firmicutes* at the phylum level [14,15,18,24]. Some studies reported the reduction in Actinobacteria and the TM7 phylum in HSCR mice [9]. These findings were consistent with a relative increase in *Proteobacteria* and Bacteroidetes with a decrease in *Firmicutes* in patients with bowel obstruction [16]. With regard to the identifiable microbes of HSCR at the genus, family, and species levels, most studies detected an increase in *Escherichia*, especially *Escherichia coli* under the phylum of *Proteobacteria* [9], an increase in *Bacteroides* [9,14] and *Tannerella* [14] under *Bacteroidetes phylum*, and a decrease in *Lactobacillus* [9,14] and *Staphylococcus* [14] under the *Firmicutes phylum*. Comparison of gut microbiota at different ages of mutant mice found a decrease in *Lactobacillus* over time and an absence of this bacterium at a late age [9,14]. In a study using piglets, the stools of HSCR piglets tended to show an increase in *Proteobacteria* at the phylum level and an increase in *Fusobacterium*, *Mogibacterium*, and *Bilophilia*, these bacteria acting as proinflammatory bacteria [15].

Studies of gut microbiota in HSCR children also reported an overabundance in *Escherichia* and *Pseudomonas* in the phylum *Proteobacteria* and an increase in *Prevotella* and *Actinomyces* under Bacteroidetes and Actinobacteria phyla, respectively [18], while *Lactobacilli*, *Lachnospiraceae*, and *Ruminococcaceae* in the phylum *Firmicutes* were significantly decreased in HSCR children after surgical treatment [18,25]. With regard to post colorectal surgery in colorectal cancer, *Proteobacteria* especially *Klebsiella*. were significantly increased after colorectal surgery. An overabundance of *Proteobacteria* was also found in several disorders. A previous review article from Na-Ri Shin et al. demonstrated that an increase in *Proteobacteria* was a microbial signature of gut dysbiosis, and the inability to control the expansion of this bacteria might predispose inflammation and the invasion by exogenous pathogens [26]. All these findings are illustrated in Table 2.

## 5. The Diversity of Gut Microbiota in Hirschsprung Disease with and without Enterocolitis: Evidence from Animal to Clinical Studies

Owing to its importance in postoperative complications and clinical outcomes, several researchers studied and reported the alterations in gut microbiota in HAEC episodes. Cheng Z et al. reported clinical enterocolitis or HAEC in HSCR mice after microsurgical resection of the aganglionic segment [6]. The alpha diversity of HAEC mice significantly reduced when compared with wild-type mice, and tended to reduce when compared with HSCR mice without HAEC [6]. Several pieces of evidence reported alterations in gut microbiota in HAEC patients. Most of this evidence was cross-sectional in nature, comparing gut microbiota between HSCR patients with clinical enterocolitis and those without enterocolitis, by measuring gut microbiota at the time of the surgery and in the postoperative period [5,18,27,28]. Two continuous studies showed a significant difference in the beta diversity of gut microbiota between patients presenting with and without HAEC during the operative time [27,28]. Both studies also detected a distinct microbiome between the proximal ganglionic and distal aganglionic parts of the intestine in Hirschsprung disease [27,28]. Yan Z and his colleagues found a greater alpha diversity of gut microbiota in the distal aganglionic than that of the proximal intestine. They also found a significant increase in the alpha diversity in HAEC when compared with HSCR patients without HAEC at the time of surgery [28]. In a subgroup analysis of Li and his colleagues, the authors detected a similarity in beta diversity of gut microbiota in both proximal and distal parts of HAEC specimens [27]. The comparison of gut microbiota between HAEC patients and patients who had a history of HAEC, called “HAEC-remission”, found a similarity in the beta diversity in both groups. These findings concluded that gut microbiota in an HAEC episode could lose the site-specific microbiome presented in Hirschsprung disease. Interestingly, the disturbance in gut microbiota persisted even after the symptoms of enterocolitis were resolved [27]. There were two studies into the gut microbiota in patients who had a history of postoperative HAEC compared with HSCR patients without a history of HAEC after definitive surgery, which implied that all the patients would not have the symptoms of an intestinal obstruction. Frykman PK et al. showed that the bacterial microbiome significantly increased in the enterocolitis group, while the diversity of the fungal mycobiome significantly reduced with an expansion of mycotic pathogens such as *Candida albicans*, especially in severe HAEC [5]. Moreover, the authors observed the depletion of *Malassezia* and *Saccharomyces* species in the patients who had a history of HAEC after surgery [5]. There was less evidence to determine the mechanism of the overabundance of Candida due to limited information regarding long-term antibiotic treatment which usually caused the fungal expansion. However, this study suggested there was a role of antifungal therapy in selected HAEC patients [5]. In addition, several clinicians found the co-incidence of prenatal cytomegalovirus infection with HSCR patients. Some studies proposed the controversial association of prenatal cytomegalovirus infection and the development of HSCR symptoms [29,30], but no study found a direct association between this virus and the development of HAEC. All these of findings suggest that the contribution to enterocolitis by disturbance of the gut microbiota may extend beyond bacteria [5]. On the other hand, another study found a decrease in the richness of the microbiota in HAEC patients after definitive surgery [18]. The possible explanation for this inconsistency between studies may be due to the difference in the ages of participants in each study and antibiotic usage. Regarding ages of participants, the median age of patients was 2.7 years old in the HAEC group in a study by Frykman PK and colleagues, while the median age in the HAEC group in a study by Neuvonen et al. was 12 years old. Regarding antibiotic usage, only three patients out of nine in Frykman’s study received antibiotics for 2 months prior to the stool collection, while nearly all patients in Neuvonen’s study used prophylactic oral antibiotics.

There were two longitudinal studies into intestinal tissue microbiota at the time of surgery and the development of postoperative HAEC. Tang W et al. studied mucosal gut microbiota in Chinese HCSR patients at the time of surgery, then followed up those patients for the potential development of clinical enterocolitis. The results found a decrease in alpha diversity of patients who developed HAEC, known as “HAEC potential patients” [7]. However, the study by Arbizu RA in American HSCR patients showed an increase in the alpha diversity of aganglionic colonic tissue microbiota at the time of surgery in the patients who developed postoperative HAEC [31]. Although the purpose of the two studies was to identify the characteristic of gut microbiota in the patients who would develop postoperative enterocolitis or HAEC potential patients, there were differences in specimen collection for the investigation of gut microbiota between the studies. Tang’s study used fresh mucosal specimens from the dilated colon to represent normal innervated tissue, while Arbizu’ study used formalin- and paraffin-fixed tissue from the aganglionic segment to study gut microbiota. Therefore, the differences in results of gut microbiota between the studies [7,31] may be due to the differences in collection and preparation of the specimens.

There was one self-control study of fecal microbiota between HAEC and normal episodes in same patient after surgery [32]. The authors found a significant difference in the beta diversity of fecal microbiota between the time of HAEC and in normal episodes [32]. Due to the limited number of studies and different design of each study, a direct comparison of the diversity of gut microbiota from each study has been impossible. Therefore, standardization of technique needs to be adhered to in any future studies which compare the changes in gut microbiota at different time points, such as preoperative, peri-operative, and postoperative periods, to discover the association between gut microbiota and the development of HAEC. All these findings are illustrated in Table 3.

Due to the variation in design in the studies, we could not directly compare the concordant or discordant results regarding the microbiota in HAEC patients. However, we divided those previous studies into three groups with regard to timing of specimen collection. The first group was the studies that collected specimens at the time of surgery and compared the microbiota in patients with and without a history of HAEC [27,28]. The authors did not always clearly identify that the patients in the HAEC group were in an acute phase of HAEC at the time of surgery. Both studies of this group proposed that the differences in beta diversity between patients with and without enterocolitis were observed [27,28]. The second group were in the postoperative condition, which compared the microbiota in patients with and without a history of HAEC, without information regarding acute symptoms [5,18]. This second group showed discordant diversity of postoperative gut microbiota between patients with and without history of HAEC [5,18]. The third group involved studies investigating gut microbiota at the time of surgery without clinical HAEC to predict postoperative HAEC [7,31]. Information regarding the history and duration of anti-biotic treatment, which could greatly affect the alteration of gut microbiome, was not clearly reported in many studies, making it impossible to determine the confounding effect of antibiotics. Until now, no study has investigated the changes in gut microbiota at different time points of treatment in HSCR patients. In addition, no direct comparison of gut microbiota in patients at an acute phase of enterocolitis and patients with a history of HAEC has been carried out.

## 6. The Alterations in Gut Microbiota Taxa in Hirschsprung Disease with and without Enterocolitis: Evidence from Animal to Clinical Studies

HSCR mice with enterocolitis after microsurgery showed a significant *increase* in *Akkermansia* in the phylum Verrucomicrobia with a decrease in *Bacteroides* (phylum: Bacteroidetes) and *Clostridium* XIVa (phylum: *Firmicutes*) [6]. Previous studies [6,33,34] demonstrated that *Akkermansia*, a mucin-degrading bacteria found in rodents and humans, plays an important role in intestinal barrier function as well as having an anti-inflammatory effect on the host. However, recent studies found a reduction in Akkermansia in IBD patients [33,34]. Some studies also showed a significant increase in Akkermansia in rodents with colitis [35]. Therefore, the role of *Akkermansia* is still elusive, particularly as to whether it acts as a pathogen predisposing factor to instigate HAEC, or has a compensatory preventive role.

Cross-sectional studies in children with and without a history of HAEC found an increase in *Proteobacteria* [5,7,27,28,32], especially *Escherichia* [18,27] and *Enterobacteriaceae* [7,28], with a decrease in the phylum *Firmicutes* [5,27,28] and *Bifidobacterium* [25,32] from the phylum Actinobacteria in the patients with a history of HAEC. A predominance of the phylum *Proteobacteria* was still detected in patients with a history of HAEC, when compared with patients without enterocolitis episodes. This finding emphasized that the expansion of *Proteobacteria* was mainly associated with gut dysbiosis, which can lead to many diseases. In addition, several studies reported a correlation between the susceptibility to colitis and the overabundance of *Proteobacteria* [26,36]. For example: (1) The genetically susceptible colitis mice, lacking Toll-like receptor (TRL)-5, exhibited a disturbance of colonic mucous layers and delayed clearance of infectious bacteria, leading to a dominance of *Proteobacteria*, especially from the family *Enterobacteriaceae*, resulting in a predisposition to chronic colitis [36]; (2) IBD, chronic inflammation of the intestine, which has been proved to be associated with innate and adaptive immune defects [37]. Several IBD studies also demonstrated an increase in *Proteobacteria* with a depletion of *Firmicutes*, when compared with normal controls [38,39,40]; (3) Tash-T mutant mice or HSCR mice showed a dysregulated activity of Toll-like receptors (TLRs) at the surface of enteric neurons in the mutant intestine. Those mice also showed an increase in *Proteobacteria* with a depletion of *Firmicutes* in HSCR mice, when compared with wild type [24]. All these findings in animal studies and clinical studies suggest that defects of both the mucosal barrier and immune function in the HSCR intestine lead to increased susceptibility for colonization and invasion by infectious pathogens, resulting in a predisposition to enterocolitis.

The alterations in the bacteria in the phylum Bacteroidetes were diverse. Some studies found an increase in bacteria in the *Bacteroidetes phylum* [5,28], while others reported a decrease in *Bacteroidetes* [27] and *Prevotella* [18] in patients with a history of HAEC. However, the *Bacteroides* bacteria were reduced in IBD patients [41]. Therefore, an increase in *Bacteroides* might play a protective role in HAEC, or might be associated with intestinal inflammation, rather than be specific to HAEC.

Interestingly one study reported a dominance of non-short-chain fatty acid (SCFA)-producing bacteria in patients with a history of HAEC [42]. That study also found a marked reduction in fecal SCFAs, and alteration of SCFA profiles as indicated by a decrease in proportions of acetate, when compared with butyrate acid. The SCFAs have previously been proven to be associated with mucosal defense mechanisms [11,12]. Thus, a decrease in SCFA-producing bacteria may be one of the predisposing factors for the development of enterocolitis in HSCR subjects.

The depletion of *Bifidobacterium* [25,32] and *Lactobacilli* [25] in HAEC patients have been shown when compared with HSCR patients without HAEC and healthy children, respectively. *Bifidobacterium* and *Lactobacilli* have been used as probiotics. Both types of bacteria promoted gut defense mechanisms via strengthening intestinal barrier function and immune systems [25,43,44]. Those bacteria also increased in exclusively breast-fed infants. Some studies demonstrated a significant reduction in pre and postoperative HAEC with a decrease in Gram-negative bacteria in exclusively breast-fed patients [7]. Therefore, the depletion of *Bifidobacterium* and *Lactobacilli* might lead to a decrease in intestinal defense mechanisms and be a predisposing factor in the development of HAEC [25].

Prospective studies into colonic microbiota at the time of surgery in postoperative HSCR participants showed an increase in *Enterobacteriaceae* and *Escherichia* from the phylum *Proteobacteria* [7,31] and an in phylum *Firmicutes* [31] in HAEC potential patients. Tang and colleagues also detected a total of 131-OTUs of microbiomes that had differences in potential postoperative HAEC specimens in comparison with specimens of non-HAEC patients. The authors also identified 21-OTUs bacteria, including nine OTUs in the *Enterobacteriaceae* family, to predict postoperative HAEC with 85% accuracy [7]. This finding is potentially useful in predicting the risk of postoperative HAEC in the patients in whom these colonic microbiomes were detected at the time of surgery. The findings of the study by Arbizu differed from the study mentioned previously that reported a tendency for *Firmicutes* bacteria to decrease in patients with a history of HAEC. The possible explanation may be that the analysis of gut microbiota in the later study was carried out from the aganglionic segment of the colon at the time of surgery which was free from enterocolitis [31]. All these findings are illustrated in Table 4.

## 7. The Alterations in Gut Microbiota following Interventions of Enterocolitis in HSCR: Evidence from Animal to Clinical Studies

Many researchers tried to discover the interventions needed to prevent the occurrence or alleviate the severity of enterocolitis. In clinical practice, cyclic antibiotic usage has been proposed with the aim of reducing the occurrence and severity of HAEC in HSCR patients. Since the hypothesis of bacterial overgrowth was believed to be a cause of HAEC, Rintala and Lindahl suggested an empirical usage of metronidazole and norfloxacin for antibiotic prophylaxis against HAEC [45]. However, a recent study suggested the prophylactic usage of antibiotics might increase the risk of resistant organisms without conferring any benefit with regard to the prevention of enterocolitis [46]. To confirm the effect of antibiotics on gut microbiota, Toure AM, and colleagues studied the composition of the gut microbiota of TashT knock-out mice and wild-type mice after administration of oral board spectrum antibiotics. After prolonged oral antibiotic usage, the gut microbiota had decreased in quantity with a reduction in the *Firmicutes* and Bacteroidetes phyla in both HSCR and wild-type mice [24]. These results suggested that prolonged usage of antibiotics might cause dysbiosis of gut microbiota without significant benefit regarding the prevention of HAEC development.

Probiotics, living microorganisms, have been proven to enhance outcomes of treatment in many disorders, especially in inflammatory conditions. Several studies tried to determine the effect of probiotics in the prevention of HAEC in HSCR patients [46,47]. Two systematic reviews and meta-analyses [46,47], including two prospective randomized control trials during the periods 1992–2017 [48,49], demonstrated that prophylactic probiotics have no benefit in a significant reduction in the incidence of HAEC. However, the recent prospective randomized controlled trial study by Wang X. et al. in 60 HSCR patients found probiotics not only reduced the incidence of HAEC, but also decreased the severity of HAEC [49]. To confirm the role of probiotics in the gut microbiota in HSCR patients, Singer G’s study compared the stools of post-surgical HSCR patients in the period after continuous treatment with oral probiotics containing *Lactobacillus* and *Bifidobacterium* spp. with the stools at the free probiotic periods. The authors detected a decrease in HAEC symptoms from 18% at the time of the free probiotic periods to 14% in the three-month period of probiotics usage [32]. This study also found a significant increase in the alpha diversity of fecal microbiota and a significant difference in microbiota composition, including the presence of the bacteria not being included in the probiotic sachet, when compared with the probiotic-free period. These findings suggested that probiotic treatment might significantly improve gut dysbiosis contributing to the improvement of the severity and development of HAEC [32]. All these findings are summarized in Table 5. However, only one study directly determined the effect of probiotics in gut microbiota. This study [32] was the self-control study in only one patient. To answer the hypothesis that probiotics improve the dysbiosis of gut microbiota and prevent the development of HAEC, randomized control trial studies or longitudinal observations are strongly recommended in the future.

## 8. Conclusions and Future Perspectives

The important evidence from both animal and clinical studies is that the gut microbiome has been obviously different between normal, HSCR, and HAEC subjects and also changes over time. In mice studies, the diversity of the gut microbiota increased over time with age when compared with normal subjects, and decreased after surgical correction. Postoperative HAEC mice had lower microbiota diversity when compared with non-HAEC and wild-type mice. On the contrary, according to the limited clinical studies in HSCR and normal children, the evidence only showed that the diversity of the microbiota decreased in HSCR patients after surgery. A direct comparison of the diversity of gut microbiota in patients with a history of HAEC and HAEC-potential patients from each study has been impossible owing to different designs and method of specimen collection in each study.

**Figure 1:**
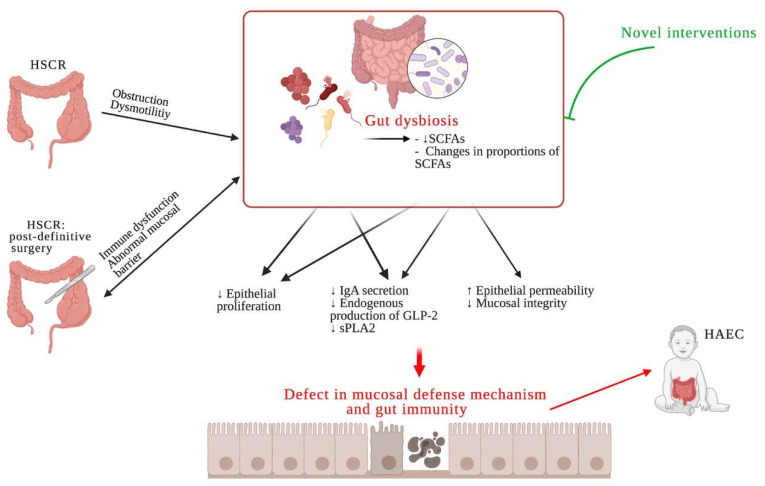
Gut microbiota dysbiosis in Hirschsprung disease and Hirschsprung-associated enterocolitis (Figure description as Appendix A).

Previous evidence demonstrated the existence of gut microbiota dysbiosis in Hirschsprung disease with an unclear etiology. Gut dysbiosis in HSCR might be due to a delayed transit time from the intestinal obstruction or dysmotility, but the dysbiosis still occurs even after definitive surgery to release the obstruction. The abnormal mucosal barrier and immune function in the HSCR intestine may be the factors contributing to gut dysbiosis. Microbiota dysbiosis in HSCR causes defects in the mucosal defense mechanism and gut immunity via many possible pathways (see Figure 1) leading to an increased susceptibility for colonization and invasion by infectious pathogens resulting in a predisposition to enterocolitis. This gut dysbiosis is sustainable even after the remission of HAEC symptoms. Recently, many gaps of knowledge in the field of gut microbiota in HSCR have been recognized. These include the changes in microbiota over time in HSCR patients, the effects of the length of colonic resection, the length of aganglionic involvement, antibiotic or probiotic usage, and the actual alteration of gut microbiota or dysbiosis in HAEC potential patients. Increased understanding regarding gut microbiota dysbiosis can provide novel specific interventions to improve dysbiosis and protect against the development of enterocolitis in the future.

## Figures and Tables

**Table 1:** The comparison of the diversity in gut microbiota between subjects with Hirschsprung disease (HSCR) and healthy controls: Evidence from animal to clinical studies.

HSCR Model/Age (N)	Control/Age (N)	Specimens/Time of Collection	Methods	Diversity			Interpretation	Ref.
				Alpha	Type of Analysis	Beta		
EdnrB-null mice/Early age [P16–18] (*n* = 6) Late age (P21–24) (*n* = 4)	EdnrB-het mice/Early age [P16–18] (*n* = 8) Late age [P21–24] (*n* = 11)	Cecal content/early and late ages	16S rRNA sequencing	Early: ↑ Late: ↑	Shannon	Significantly differed between the late control group and others.	Alpha diversity in Hirschsprung mice increased continuously over time, while the diversity was decreased in normal controls.	[9]
EdnrB null mice/P7, 20, 24 (*n* = 13)	WT/P7, 20, 24 (*n* = 13)	Fecal and colonic mucosa/P7,20,24 days	16S rRNA sequencing	Colon ↑ P7 ↔ P20 and P24 ↑ with age Fecal: ↑ P7 ↑ P20 ↔ P24	OTUs Shannon	Differences seen between both groups. Differences seen between all postnatal ages in both fecal and colonic specimens.	The alpha diversity in early age mutant mice was increased with age and greater than WT, while both fecal and colonic specimens had different beta diversity between both mutant-WT and all postnatal age groups.	[14]
0.5% BAC serosal rectosigmoid soaked piglets/P26 (*n* = 7)	saline soaked piglets/P26 (*n* = 5)	Rectosigmoid content/P26 days	16S rRNA sequencing (V3–4)	↔	Chao1 Shannon	Significant difference between BAC and control piglets.	There was no significant difference in the alpha diversity but a difference in the beta diversity between BAC and control piglets.	[15]
Ednrb-/- mice/PO 0, 14, and 28 days (*n* = 10)	WT mice/PO 0, 14, and 28 days (*n* = 8)	Stool/PO 0,14, and 28 days	16S rRNA sequencing (V4–5)	↓	Shannon	Difference between mutant and WT. Difference between PO 0, 14 and 28 in both mutant and WT groups.	After surgery, the alpha diversity was significantly reduced in mutant mice, while the beta diversity differed between mutant-WT and in all postoperative periods. Surgery itself might alter microbiota.	[6]
HSCR patients/3–25 yrs (*n* = 34)	non-HSCR/2–7 yrs (*n* = 141)	Stool/post definitive surgery.	16S rDNA sequencing (V3–4)	↓ richness		N/A	The richness of gut microbiota in HSCR patients was significantly reduced after surgery.	[18]

Abbreviations: EdnrB: Endothelin receptor type B; het: heterozygous; P: postnatal day; rRNA: ribosomal ribonucleic acid; WT: wild type; OTUs: Operational taxonomic units; BAC: benzalkonium chloride solution; PO d: postoperative day; HSCR: Hirschsprung disease; rDNA: ribosomal deoxyribonucleic acid. ↑: increase or higher in diversity, ↓: decrease or lower in diversity, ↔: no change or equal in diversity.

**Table 2:** The changes of taxa in gut microbiota between subjects with Hirschsprung disease (HSCR) and healthy subjects: Evidence from animal to clinical studies.

HSCR Model/Age (N)	Control/Age (N)	Specimens/Time of Collection	Methods	Taxonomy					Interpretation	Ref.
				Phylum	Genus, Family, Species					
					*Proteobacteria*	*Firmicutes*	*Bacteroides*	Others		
EdnrB-null mice/Early age [P16–18] (*n* = 6) Late age [P21–24] (*n* = 4)	EdnrB-het mice/Early age [P16–18] (*n* = 8) Late age [P21–24] (*n* = 11)	Cecal content/early and late ages	16S rRNA sequencing	Early: N/A Late: ↓Actinobacteria ↓TM7	Late: ↑*Escherichia*	Early: ↔*Lactobacillus* Late: ↑*Clostridium* ↓*Lactobacillus* (↓overtime)	Late: ↑*Bacteroides*		The mutant mice showed a decrease in *Lactobacillus* over time with an increase in a possible pathogen (*Escherichia coli*), leading to EC.	[9]
EdnrB-null mice/Early age [P7] Late age [P20,24] (*n* = 13)	WT mice/Early age [P7] Late age [P20,24] (*n* = 13)	Fecal and colonic mucosal microbiota/Early age [P7] Late age [P20,24]	16S rRNA sequencing	Colon (Early): N/A Colon (Late): ↑*Proteobacteria* ↑↑Bacteroidetes ↓↓*Firmicutes* Fecal (Late): ↑*Proteobacteria* ↑↑Bacteroidetes ↓↓Firmicute		Colon (Early): ↑*Staphylococcus* (*S. xylosus*) ↑*Lactobacillus* ↑*Coprobacillus* ↓Clostridium Colon (Late): ↑*Clostridium* ↑↑*Coprobacillus* Absent *Staphylococcus* Absent *Lactobacillus* Fecal (Late): ↓*Lactobacillus* ↓*Staphylococcus* (*S*. *Xylosus*)	Colon (Early): ↑*Bacteroides* Colon (Late): ↑↑*Bacteroides* Fecal (Late): ↑*Tannerella*		The mutant fecal and colonic specimens increased in Bacteroidetes and *Proteobacteria* but decreased in *Firmicutes* esp. *Lactobacillus* and *Staphylococcus* over time and changed with age.	[14]
0.5% BAC serosal rectosigmoid soaked piglets/P5 (*n* = 7)	saline soaked piglets/P5 (*n* = 5)	Rectosigmoid content/P26 days	16S rRNA sequencing (V3–4)	Tendency ↑*Proteobacteria*	↑*Bilophilia*	↑*Mogibacterium*		↑*Fusobacterium* (Fusobacteria)	BAC piglet stools showed a tendency to increase in *Proteobacteria* at the phylum level and increase in Fusobacterium, *Mogibacterium*, Bilophilia (proinflammatory bacteria).	[15]
TashT Tg/Tg, mice/P21–22 (*n* = 9) and Holstein Tg/Tg mice/P21–22 (*n* = 3)	WT mice/P21–22 (*n* = 6)	fecal sample from the colon/weaning age	16S rRNA sequencing (V5–6)	↑↑*Proteobacteria* ↑Deferribacteres ↓↓*Firmicutes*					The mutant mice feces significantly increased in *Proteobacteria* and decreased in *Firmicutes* at the phylum level.	[24]
HSCR patients/3–25 yrs. (*n* = 34)	Non-HSCR patients/3–25 yrs. (*n* = 141)	Stool/post definite surgery	16S rDNA sequencing (V3–4)	↑*Proteobacteria*	↑*Escherichia* ↑*Pseudomonas*	↑*Dialister* ↑*Bacilli* ↓*Ruminococcaceae* ↓*Lachnospiraceae* ↓*Lactobacilli*	↑*Prevotella* ↓*Bacteroidales*	↑*Actinomyces* (Actinobacteria)	After surgery, HSCR stools showed an expansion of *Proteobacteria* at the phylum level and Enterobacteria and Bacilli at order level. While *Lactobacilli* decreased significantly.	[18]
HSCR patients/2 wks–2 yrs. (*n* = 20)	Non-HSCR patients/2 wks–2 yrs. (*n* = 15)	Stool	16S rRNA real-time PCR			↓*Lactobacilli*			*Lactobacilli* significantly decreased in HSCR specimens.	[25]

Abbreviations: EdnrB: Endothelin receptor type B; het: heterozygous; P: postnatal day; rRNA: ribosomal ribonucleic acid; TM7: Saccharibacteria; EC: enterocolitis; WT: wild type; esp.: especially; BAC: benzalkonium chloride solution; rDNA: ribosomal deoxyribonucleic acid; HSCR: Hirschsprung disease; PCR: polymerase chain reaction. ↑: increase or higher, ↓: decrease or lower, ↔: no change or equal.

**Table 3:** The diversity in gut microbiota in Hirschsprung disease with and without enterocolitis: evidence from animal to clinical studies.

HSCR with EC Model/Age (N)	HSCR without EC Model/Age (N)	Specimens/Time of Collection	Methods	Diversity			Other Findings	Interpretation	Ref.
				Alpha	Type of Analysis	Beta			
HAEC patients/2, 6 mo. (*n* = 2)	HSCR patients/7, 12 mo. (*n* = 2)	Intestinal content from different sections/during surgery	16S rDNA sequencing (V1–3)	↑	OTUs	Difference	HSCR had greater diversity in distal than proximal samples. HAEC had greater diversity in proximal than distal samples.	HAEC samples increased the alpha diversity, while both HSCR-HAEC had a difference of microbiome between proximal (ganglionic) and distal (aganglionic) parts of the intestine.	[28]
HAEC/HAEC-R patients/10 d.–2 yrs. (*n* = 5/3)	HSCR patients/10 d.–2 yrs. (*n* = 5)	Intestinal contents from difference sites/during surgery	16S rRNA sequencing (V4)	N/A		Difference (HSCR-HAEC) Similarity (HAEC-HAEC-R)	HSCR showed distinct microbiomes between the proximal-distal intestine. Both HAEC and HAEC-R specimens showed no different microbiota in sites.	HAEC specimens found a loss of a site-specific microbiome of HSCR and HAEC-R had persistent disturbance similar to HAEC even when symptoms of EC were resolved.	[27]
HAEC patients/5 mo.–8 yrs. (*n* = 9)	HSCR patients/5 mo.–8 yrs. (*n* = 9)	Stools/after definitive surgery	16S rRNA sequencing (V1–4)	↑ ↓ (mycobiome)	Shannon OTUs	N/A		The stools of HSCR patients had an increased alpha diversity in the microbiome but a decreased alpha diversity in the mycobiome.	[5]
HAEC patients/3–25 yrs. (*n* = 26)	HSCR patients/3–25 yrs. (*n* = 8)	Stools/post definitive surgery	16S rDNA sequencing (V3–4)	↓ richness		N/A		The loss of richness in the microbiota led to an increase in vulnerability to colonizing pathogens in HAEC.	[18]
HAEC episodes/3 yrs. (*n* = 3)	Non-HAEC episodes/3 yrs. (*n* = 3)	Self-comparisons of stools	16S rRNA sequencing	↔	Chao1	Difference		There was a difference in Beta diversity between HAEC and non-HAEC periods.	[32]
post-op HAEC patients/mostly <3 mo. (*n* = 25)	HSCR patients/mostly <3 mo. (*n* = 50)	Mucosa at edge of dilated segment close to normal/at time of surgery	16S rRNA sequencing (V4)	↓	OTUs Chao1 Shannon Simpson PD	Difference		In postoperative HAEC patients, a decrease in the alpha diversity of microbiota in a mucosal specimen at the time of surgery was found.	[7]
post-op HAEC patients/mostly <1 mo. (*n* = 4)	HSCR patients/mostly <1 mo. (*n* = 4)	Aganglionic colonic tissue formalin and paraffin fixation/at time of surgery	16S rDNA sequencing (V3,4)	↑	observed alpha diversity	No significant difference		In postoperative HAEC patients or HAEC potential patients, an increase in the alpha diversity of microbiota in aganglionic colon specimens at the time of surgery was found without significant difference in the beta diversity between HSCR and potential HAEC groups.	[31]
Ednrb-/- mice with HAEC/3–6 wks. (*n* = 6)	WT mice/3–6 wks. (*n* = 4)	Stool/PO d0,14,28	16S rRNA sequencing (V4–5)	↓	OTUs Chao1 Shannon Simpson PD	N/A		In a potential HAEC model, a decrease in the alpha diversity of microbiota in stools at the time of surgery was found.	[6]

Abbreviations: EC: enterocolitis; HAEC: Hirschsprung-associated enterocolitis; HSCR: Hirschsprung disease; rDNA: ribosomal deoxyribonucleic acid; OTUs: Operational taxonomic units; HAEC-R: Hirschsprung-associated enterocolitis remission; rRNA: ribosomal ribonucleic acid; EdnrB: Endothelin receptor type B; PO: postoperative day. ↑: increase or higher in diversity, ↓: decrease or lower in diversity, ↔: no change or equal in diversity.

**Table 4:** The alterations of gut microbiota taxa in Hirschsprung’s disease with and without enterocolitis: evidence from animal to clinical studies.

HSCR with EC Model/Age (N)	HSCR without EC Model/Age (N)	Specimens/Time of Collection	Methods	Taxonomy					Interpretation	Ref.
				Phylum	Genus, Family, Species					
					*Proteobacteria*	*Firmicutes*	*Bacteroides*	Others		
HAEC patients/2, 6 mo. (*n* = 2)	HSCR patients/7, 12 mo. (*n* = 2)	Intestinal content from different sections/during surgery	16S rDNA sequencing (V1–3)	↑*Proteobacteria* ↓*Firmicutes*	↑*Enterobacteriaceae* ↓*Acinetobacter*	↑*Enterococcus*	↑*Bacteroides*	↓*Fusobacterium* (Fusobacteria) ↓*Eukaryota*	HAEC specimens had an increase in *Proteobacteria* but a decrease in *Firmicutes*.	[28]
HAEC/HAEC-R patients/10 d.–2 yrs. (*n* = 5/3)	HSCR patients/10 d.–2 yrs. (*n* = 5)	Intestinal contents from different sites/during Surgery	16S rRNA sequencing (V4)	↑↑*Proteobacteria* ↓↓ Bacteroidetes ↓*Firmicutes*	↑↑*Escherichia* ↓*Acinetobacter*	↓*Veillonella*	↓*Bacteroidete*		An increase in *Proteobacteria* and a deficiency in *Bacteroides*-*Firmicutes* might be associated with HAEC.	[27]
HAEC patients/5 mo.–8 yrs. (*n* = 9)	HSCR patients/5 mo.–8 yrs. (*n* = 9)	Stool/after complete definitive surgery	16S rRNA sequencing (V1–4)	↑*Proteobacteria* ↑Bacteroidetes ↓*Firmicutes* ↓Verrucomicrobia		No significant difference		↑*C*. *albican* (mycobiome) ↓*C. tropicalis* (mycobiome) ↓*Malassezia* (mycobiome) ↓*Saccharomyces* sp. (mycobiome)	An increase in *Proteobacteria*, a decrease in *Firmicutes*, and an increase in the pathologic mycobiome were detected in the HAEC stools.	[5]
History of HAEC patients/3 mo–8 yrs. (*n* = 9)	HSCR patients/3 mo–8 yrs. (*n* = 9)	Stool/after definitive surgery	16S rRNA sequencing (V1–4)	Dominated by non-SCFA-producing bacteria					The microbiota changed to be dominated by non-SCFA-producing bacteria in HAEC.	[42]
HAEC patients/3–25 yrs. (*n* = 26)	HSCR patients/3–25 yrs. (*n* = 8)	Stool/post definitive surgery	16S rDNA sequencing (V3–4)		↑↑*Escherichia* ↑↑*Shigella* ↑*Proteobacteria*	↑*Lactococcus* ↑*Lactobacillus* ↓↓*Clostridia* ↓*Oscillospira* ↓*Holdemania*	↓↓*Prevotella*		HAEC increased vulnerability to colonization by pathogens.	[18]
HAEC patients/2 wks–2 yrs. (*n* = 10)	Non-HAEC patients/2 wks–2 yrs. (*n* = 20)	Stool	16S rRNA real time PCR					↓*Bifidobacterium* (Actinobacteria)	The scarcity of *Bifidobacterium* might be a predisposing cause of EC.	[25]
HAEC episodes/3 yrs. (*n* = 3)	Non-HAEC episodes/3 yrs. (*n* = 3)	Self-comparison of stool	16S rRNA sequencing	↑*Proteobacteria* ↑Bacteroidetes ↑Cyanobacteria ↓Actinobacteria				↓*Bifidobacterium* (Actinobacteria)	HAEC stools showed increased *Proteobacteria*, Bacteroidetes, Cyanobacteria, and significantly decreased Actinobacteria esp. *Bifidobacterium*.	[32]
post-op HAEC patients/mostly <3 mo. (*n* = 25)	HSCR patients/mostly <3 mo. (*n* = 50)	Mucosa at dilated segment close to normal/at time of surgery	16S rRNA sequencing (V4)		↑*Enterobacteriaceae*			↑*Koribactereceae* (Acidobacteria)	An increase in *Enterobacteriaceae* was detected in HAEC potential patients.	[7]
post-op HAEC patients/mostly <1 mo. (*n* = 4)	HSCR patients/mostly <1 mo. (*n* = 4)	Aganglionic colonic tissue formalin and paraffin fixation/at time of surgery	16S rDNA sequencing (V3,4)	↑↑*Firmicutes* ↑Bacteroidetes ↑Cyanobacteria	↑*Escherichia*	↑*Dolosigranulum* ↑*Streptococcus* ↑*Roseburia* ↑Enterococcus		↑*Propionibacterium* (Actinobacteria)	An increase in bacteria in phylum *Firmicutes*, especially *Dolosigranulum, Streptococcus,* and *Roseburia* was detected in postoperative HAEC patients.	[31]
Ednrb-/- mice with HAEC/3–6 wks. (*n* = 6)	Ednrb-/- mice without HAEC/3–6 wks. (*n* = 4)	Stool/PO d0,14,28	16S rRNA sequencing (V4–5)	↑↑Verrucomicrobia ↓Bacteroidetes ↔*Firmicutes*		↓*Clostridium XIVa*	↓*Bacteroidetes* ↓*Dysgonomas*	↑↑*Akkermansia* (Verrucomicrobia)	Markedly increased *Akkermansia* and decreased Bacteroidetes might be associated with EC.	[6]

Abbreviations: EC: enterocolitis; HAEC: Hirschsprung-associated enterocolitis; HSCR: Hirschsprung disease; rDNA: ribosomal deoxyribonucleic acid; HAEC-R: Hirschsprung-associated enterocolitis remission; rRNA: ribosomal ribonucleic acid; SCFA: short-chain fatty acid; PCR: polymerase chain reaction; EdnrB: Endothelin receptor type B; PO: postoperative day. ↑: increase or higher, ↓: decrease or lower, ↔: no change or equal.

**Table 5:** The alterations in gut microbiota following interventions: evidence from animal to clinical studies.

Model/Age (N)	Control/Age (N)	Specimens/Time of Collection	Intervention	Methods	Diversity			Taxonomy	Interpretation	Ref.
					Alpha	Beta	Phylum	Genus, Family, Species		
TashT Tg/Tg mice ^a^/P21–22 (*n* = 3) WT/P21–22 (*n* = 3) + ABO	TashT Tg/Tg mice ^a^/P21–22 (*n* = 3) WT/P21–22 (*n* = 3) − ABO	fecal sample in colon /P21–22	Oral broad spectrum ABO ^b^ prenatal 10 day—P36	16S rRNA sequencing (V5–6)	↓	N/A	↑↑Tenericutes ↓*Firmicutes* ↓Bacteroidetes	↑↑↑*Mycoplasma* (Tenericutes) ↑*Enterobacter ales* (*Proteobacteria*) ↓↓*Clostridiales* (*Firmicutes*) ↓↓*Bacteroidetes* (Bacteroidetes) ↓↓*Campylobacterales* (*Proteobacteria*) ↓↓*Burkholderiales* (*Proteobacteria*)	Antibiotics decreased fecal microbiota similarly in both mutant and WT, causing dysbiosis.	[24]
HAEC episode/3 yrs. (*n* = 6) Non-HAEC episode/3 yrs. (*n* = 8) + Probiotics	HAEC episode/3 yrs. (*n* = 6) Non-HAEC episode/3 yrs. (*n* = 8) − Probiotics	self-comparisons of stool	Probiotic ^c^ period 3 mo.	16S rRNA sequencing	↑	Significant difference	↑Bacteroidetes	↑*Bifidobacterium* (Actinobacteria) ↑*Streptococcus* (*Firmicutes*) ↓*Rikenellaceae* (*Bacteroides*) ↓*Pseudobutyrivibrio* (*Firmicutes*) ↓*Blautia* (*Firmicutes*) ↓*Lachnospiraceae* (*Firmicutes*)	Probiotic significantly improved gut-dysbiosis in HAEC patients.	[32]

Abbreviations: ABO: antibiotic; P: postnatal day; WT: wild type; rRNA: ribosomal ribonucleic acid; mcl: microliter; HAEC: Hirschsprung-associated enterocolitis; CFU: colony forming unit. ^a^ Mice: TashT Tg/Tg, model *n* = 6 (oral ABO *n* = 3, non-oral ABO *n* = 3) and wild type *n* = 6 (oral ABO *n* = 3, non-oral ABO *n* = 3). ^b^ oral board spectrum antibiotics: vancomycin, ampicillin, neomycin, metronidazole. ^c^ Probiotic: 1 sachet of OMNi-BiOtiC^®^ PANDA contains Lactococcus lactis W58, *Bifidobacterium bifidum* W23, and *Bifidobacterium lactis* W52 (total of 3 × 10^9^ CFU/sachet) in the morning and 1 sachet of OMNi-BiOTiC^®^ 10 AAD contains *Lactobacillus* acidophilus W55, *Lactobacillus* acidophilus W37, *Lactobacillus* paracasei W72, *Lactobacillus* rhamnosus W71, *Lactobacillus* salivarius W24, *Lactobacillus* plantarum W62, *Bifidobacterium bifidum* W23, *Bifidobacterium lactis* W18, *Bifidobacterium longum* W51, and Enterococcus faecium W54 (total of 5 × 10^9^ CFU/sachet) in the evening. ↑: increase or higher, ↓: decrease or lower, ↔: no change or equal.

## Abbreviations

HSCR: Hirschsprung disease; HAEC: Hirschsprung’s associated enterocolitis; SCFAs: short-chain fatty acids; IgA: immunoglobulin A; sPLA2: secretory phospholipase A2; GLP-2: Glucagon-like peptide 2.

## Appendix A

Gut dysbiosis in HSCR might be due to delayed transit time from intestinal obstruction or dysmotility, but it still occurs even after definitive surgery to release the obstruction. The presence of an abnormal mucosal barrier and immune function in the HSCR intestine may be the factors contributing to gut dysbiosis. Microbiota dysbiosis in HSCR causes defects in the mucosal defense mechanism and gut immunity via many possible pathways including reduction and alteration in the proportion of SCFA, and reduction in secretion of IgA, activity of sPLA2 [9], and endogenous GLP-2 (gut peptide enhancing mucosal tight junctions) [50]. These mechanisms cause a decrease in epithelial proliferation and an increase in epithelial permeability leading to a reduction in mucosal integrity and an increased susceptibility for colonization and invasion by infectious pathogens predisposing to enterocolitis. This gut dysbiosis is sustainable even after remission of HAEC symptoms. Novel specific interventions will potentially improve dysbiosis and protect against the development of enterocolitis in the future.
